# Sarcoidosis With Generalized Lymphadenopathy: A Clinical Mimic of Lymphoma

**DOI:** 10.7759/cureus.78049

**Published:** 2025-01-27

**Authors:** Laxman Wagle, Anuj Timshina, Rashmita Regmi, Mustafa Abdulmahdi

**Affiliations:** 1 Internal Medicine, Ascension Saint Agnes Hospital, Baltimore, USA; 2 Internal Medicine, MedStar Franklin Square Medical Center, Baltimore, USA; 3 Internal Medicine, Patan Academy of Health Sciences, Lalitpur, NPL; 4 Nursing, Karnali Academy of Health Sciences, Jumla, NPL

**Keywords:** generalized lymphadenopathy, hilar lymphadenopathy, lymphoproliferative diseases, pulmonary sarcoidosis, sarcoidosis-lymphoma syndrome

## Abstract

Sarcoidosis is a multiorgan disease characterized by noncaseating granulomatous lesions, most commonly affecting the lungs. It typically presents with cough, dyspnea, wheezing, and chest pain. Chest X-rays often show bilateral hilar adenopathy. Extrapulmonary symptoms are also common. However, diagnosis requires ruling out other potential causes.

We present a case of a 58-year-old African-American woman with diabetes mellitus who presented with right flank pain and was found to have elevated creatinine (4.4 mg/dL) and severe hypercalcemia (15.3 mg/dL). Imaging revealed extensive lymphadenopathy, leading to suspicion of lymphoma. A comprehensive workup, including lymph node biopsy, ruled out lymphoma, and sarcoidosis was diagnosed. The patient was treated with intravenous (IV) hydration, calcitonin, and pamidronate for hypercalcemia, with subsequent normalization of calcium and improvement in creatinine levels. A lymph node biopsy confirmed granulomatous disease consistent with sarcoidosis. Flow cytometry showed no evidence of lymphoma, and the patient was started on prednisone.

Sarcoidosis presents a diagnostic challenge due to its varied presentation and the need for more sensitive and specific diagnostic tests, often leading to under-recognition and misdiagnosis. Studies have found that most patients with pulmonary sarcoidosis also have extrapulmonary involvement, with extrathoracic lymph nodes and skin being the most commonly affected sites. Sarcoidosis-lymphoma syndrome should also be considered, as the incidence of lymphoma is higher in patients with sarcoidosis. However, it can be missed due to similar presentations and a need for more awareness, impacting patient care and prognosis.

Our case emphasizes the importance of accurately diagnosing sarcoidosis and ruling out lymphoma and other lymphoproliferative diseases, given their increased incidence in sarcoidosis patients. However, treatment for lymphoma should not be initiated until the diagnosis is confirmed, as sarcoidosis can mimic lymphoma.

## Introduction

Sarcoidosis

Sarcoidosis is a rare, inflammatory disease in which clusters of immune cells, called granulomas, form in various organs throughout the body. These granulomas can disrupt normal organ function, making sarcoidosis a multiorgan system disorder. While it most commonly affects the lungs, causing symptoms like shortness of breath and persistent cough, it can also involve the skin, eyes (e.g., causing uveitis or even blindness), heart (leading to arrhythmias and cardiomyopathy), kidneys (possibly causing renal failure), and the nervous system. In severe cases, it can lead to long-term complications such as pulmonary fibrosis and pulmonary hypertension. Diagnosing sarcoidosis can be challenging due to several factors: (1) the lack of a precise, universally accepted case definition, (2) the variability in how the disease presents across different individuals, and (3) the absence of sensitive, specific diagnostic tests. As a result, sarcoidosis is often under-recognized or misdiagnosed, especially since its symptoms overlap with those of other diseases, such as infections or lymphoproliferative disorders [1]. A diagnosis usually requires the identification of characteristic granulomas in more than one organ and excluding other conditions that can cause similar granulomatous lesions [2].

Sarcoidosis-lymphoma syndrome

A particularly complex aspect of sarcoidosis is its association with lymphoma, known as sarcoidosis-lymphoma syndrome. It is crucial to differentiate sarcoidosis from "sarcoid-like reactions" caused by malignancy, immunotherapy, or other conditions in patients who do not meet the criteria for systemic sarcoidosis [3]. There are three recognized patterns of sarcoidosis-lymphoma association: (1) sarcoidosis occurring several years before lymphoma, typically after the age of 40, and often linked with Hodgkin's lymphoma; (2) concurrent sarcoidosis and lymphoma, with both diseases appearing within a year of each other; and (3) sarcoidosis developing at least a year after a lymphoma diagnosis [4-6]. This case report highlights the diagnostic challenges and underscores the importance of recognizing the potential overlap between sarcoidosis and lymphoma to avoid misdiagnosis and ensure appropriate management.

## Case presentation

A 58-year-old African-American woman with a history of diabetes mellitus presented to an urgent care facility with complaints of right flank pain. Initial laboratory investigations revealed elevated creatinine (4.4 mg/dL) and severe hypercalcemia (15.3 mg/dL), prompting a transfer to the hospital for further evaluation. The patient reported lifting heavy groceries on Christmas evening, after which she developed intermittent right-sided flank pain. She denied nausea, vomiting, diarrhea, or fever, and there was no family history of autoimmune diseases or sarcoidosis. Her only regular medication was metformin, and she did not take over-the-counter medications or calcium supplements.

Upon physical examination, the patient’s vital signs were unremarkable. She had dry mucous membranes and diffuse cervical lymphadenopathy, but the rest of the examination was otherwise unremarkable. Laboratory tests revealed normocytic anemia, elevated blood urea nitrogen, creatinine, serum calcium, and ionized calcium (Table 1). In the workup for hypercalcemia, the parathyroid hormone (PTH) level was suppressed, suggesting parathyroid-independent hypercalcemia. Vitamin D level was normal, so 1,25-dihydroxy vitamin D and PTH-related protein were sent. The 1,25-dihydroxy vitamin D level was elevated with normal PTH-related protein (Table 1). Due to the patient’s normocytic anemia and acute kidney injury, multiple myeloma was suspected, and a comprehensive myeloma panel was ordered, including urine and serum protein electrophoresis, immunofixation, and kappa/lambda ratio.

**Table 1:** Key lab findings g/dl: gram per deciliter; mg/dl: milligram per deciliter; mmol/L: millimole per liter; pg/ml: picogram per milliliter; ng/dl: nanogram per deciliter; U/L: unit per liter; pmol/L: picomole per liter; PTH: parathyroid hormone; ACE: angiotensin-converting enzyme The laboratory findings were consistent with severe hypercalcemia (calcium 15.3 mg/dL) and elevated 1,25-dihydroxy vitamin D (193 pg/mL), both of which are commonly associated with conditions such as sarcoidosis or lymphoma. The suppressed PTH level supports a diagnosis of non-parathyroid-related hypercalcemia, further narrowing the differential diagnosis

Labs	Values	Normal range
Hemoglobin	10.8 g/dl	12-15 g/dl
Calcium	15.3 mg/dl	8.4-10.2 mg/dl
Ionized calcium	1.94 mmol/L	1-1.3 mmol/L
Blood urea nitrogen (BUN)	42 mg/dl	9.8-20.1 mg/dl
Creatinine	4.4 mg/dl	0.57-1.11 mg/dl
Parathyroid hormone (PTH)	20 pg/ml	15-77 pg/ml
Vitamin D level	33.7 ng/mL	20-150 ng/mL
1,25 dihydroxy vitamin D	193 pg/mL	24.8-81.5 pg/mL
ACE level	131 U/L	14-82 U/L
PTH-related protein (PTHrP)	<2 pmol/L	<2.0 pmol/L

Imaging studies

**Figure 1:**
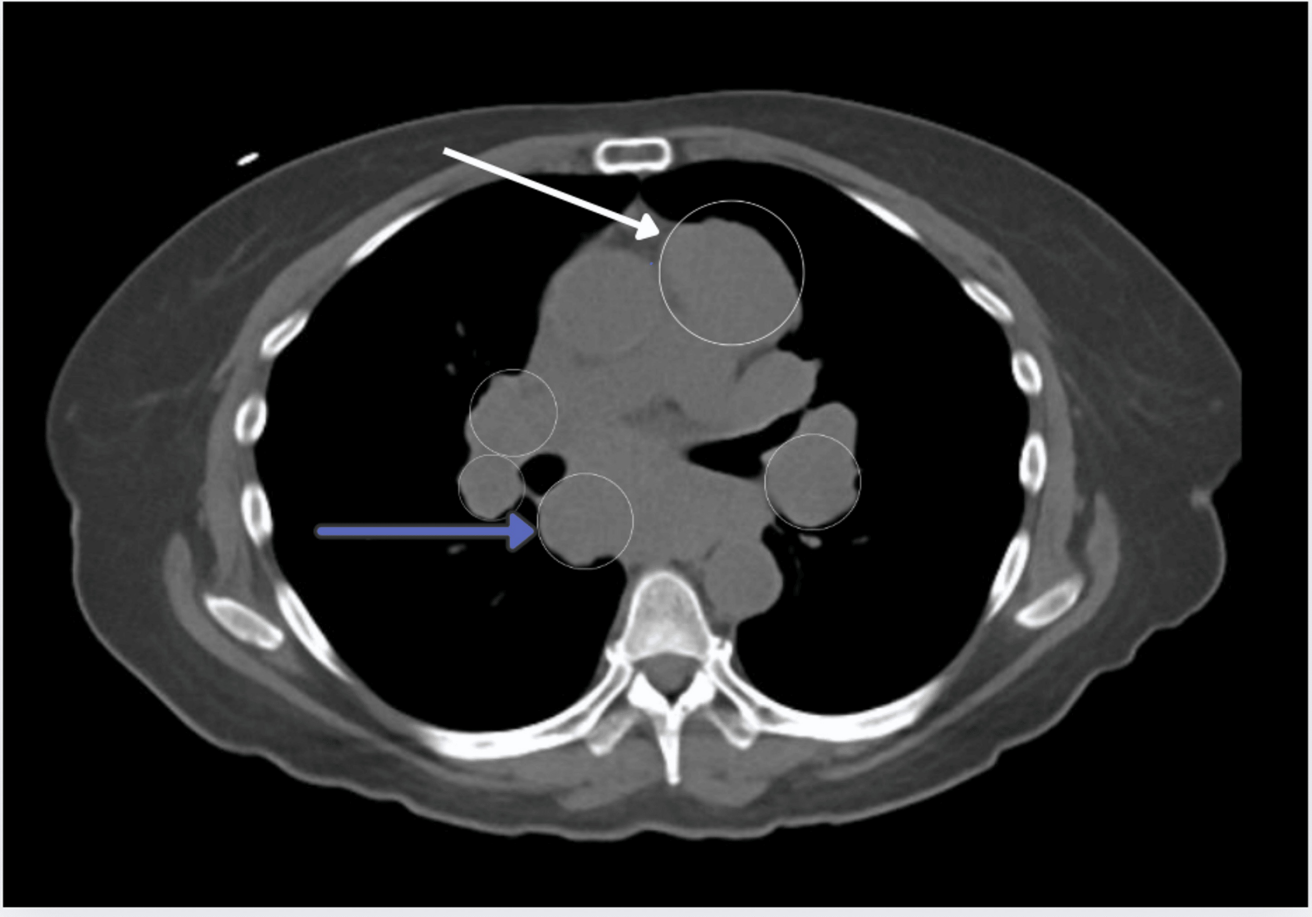
CT scan showing mediastinal (white arrow) and hilar (purple arrow) lymphadenopathy, which are suggestive of a lymphoproliferative disorder. The presence of mesenteric lymphadenopathy also raises concern for an underlying malignancy CT: computed tomography

**Figure 2:**
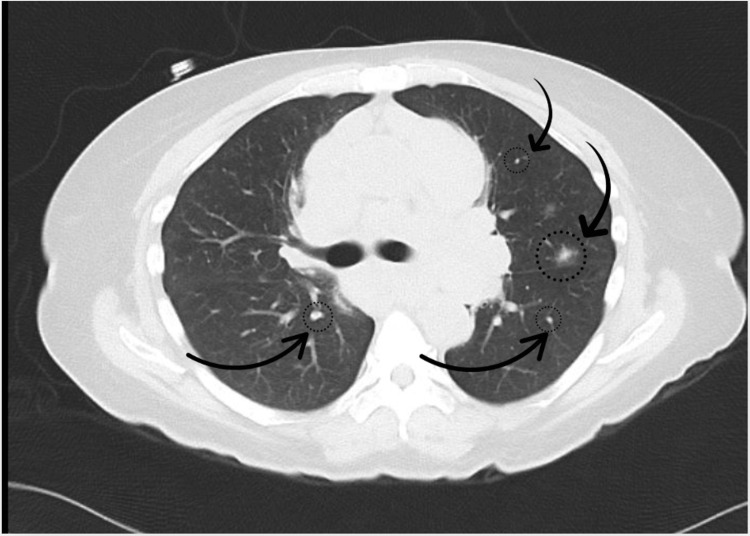
CT scan showing multiple pulmonary nodules (dotted circle with curved arrow) that may indicate metastatic disease CT: computed tomography

A computed tomography (CT) scan of the chest, abdomen, and pelvis revealed mediastinal, left hilar, and mesenteric lymphadenopathy, raising concern for lymphoma (Figure 1). Multiple pulmonary nodules were also noted (Figure 2), which could suggest metastatic disease, although the possibility of early septic emboli was considered less likely. A retroperitoneal ultrasound showed a nonobstructing 5 mm right renal calculus, but no hydronephrosis. Brain magnetic resonance imaging (MRI) demonstrated cervical, occipital, parotid, and preauricular lymphadenopathy, further suggesting metastatic disease. The brain parenchyma appeared normal. These findings raised concern for a lymphatic neoplasm, prompting a multidisciplinary discussion with oncologists and pulmonologists. A decision was made to proceed with a cervical lymph node biopsy.

Management

Given the severity of hypercalcemia, the patient was treated with aggressive intravenous hydration (250-300 mL/h), aiming for a urine output of 300 mL/h. In addition, calcitonin and pamidronate (90 mg) were administered. Zoledronic acid was not given due to her acute kidney injury. With this regimen, her calcium level normalized within four days, and her creatinine improved (Table 2). The HIV, hepatitis panel, C3, C4, antinuclear antibody (ANA), antineutrophil cytoplasmic antibody (c-ANCA), perinuclear antineutrophil cytoplasmic antibody (p-ANCA), and glomerular basement membrane (GBM) antibodies were unremarkable. Serum immunofixation revealed a polyclonal increase in immunoglobulins, and urine immunofixation showed the presence of Bence-Jones protein (kappa type) with an elevated kappa/lambda ratio (Table 2). The elevated kappa/lambda ratio and Bence-Jones protein in the urine raised initial concern for multiple myeloma. However, the hematologist clarified that the abnormal light chain levels were likely secondary to the acute kidney injury, which can cause a transient increase in free light chains, leading to skewed kappa/lambda ratios. This explains the lack of other myeloma-associated findings (e.g., M-spike and no monoclonal rise in immunoglobulin) and reassured the clinical team of the nonsignificance of this abnormality in this context.

**Table 2:** Follow-up lab findings mg/dl: milligram per deciliter Following aggressive treatment, calcium levels normalized, and kidney function improved. The light chain ratio discrepancy was deemed secondary to acute kidney injury and not clinically significant

Labs	Values	Normal range
Calcium	10 mg/dl	8.4-10.2 mg/dl
Blood urea nitrogen (BUN)	20 mg/dl	9.8-20.1 mg/dl
Creatinine	2.1 mg/dl	0.57-1.11 mg/dl
Free kappa light chain	222.2 mg/dl	3.3-19.4 mg/dl
Free lambda light chain	87 mg/dl	5.7-26.3 mg/dl
Free kappa/lambda ratio	2.55	0.26-1.65

Diagnosis and Final Management

The cervical lymph node biopsy confirmed granulomatous disease consistent with sarcoidosis. The absence of caseating necrosis and the presence of noncaseating granulomas were key histopathological findings that supported the diagnosis of sarcoidosis, distinguishing it from other granulomatous diseases such as tuberculosis. Flow cytometry showed no phenotypically abnormal populations, further excluding lymphoma.

With the diagnosis of sarcoidosis, the patient was started on a tapering dose of prednisone. She was initially given 20 mg daily for one week, followed by 10 mg daily for another week, and then 5 mg daily. The rationale for the starting dose was to address the acute inflammatory process, and the tapering duration was designed to minimize the risk of exacerbation while preventing unnecessary steroid-related side effects. She was advised to follow up with pulmonology, nephrology, and her primary care physician on an outpatient basis.

Red Flag Symptoms and Differential Diagnosis

The patient’s severe hypercalcemia, generalized lymphadenopathy, elevated angiotensin-converting enzyme (ACE) levels, and 1,25-dihydroxy vitamin D were key findings suggesting sarcoidosis. The imaging results of widespread lymphadenopathy, including mediastinal and hilar nodes, along with pulmonary nodules, initially pointed toward lymphoma. However, the combination of these findings can also be seen in sarcoidosis, highlighting the need for careful differential diagnosis. The elevated ACE and 1,25-dihydroxy vitamin D levels are significant in sarcoidosis, as these markers are typically elevated in active disease due to granulomatous inflammation. Additionally, the imaging findings of bilateral hilar lymphadenopathy and pulmonary involvement align with the classic pattern seen in sarcoidosis.

## Discussion

Sarcoidosis presents a challenge for diagnosis due to its varied presentation and the absence of sensitive and specific diagnostic tests, leading to under-recognition and misdiagnosis [1]. Our case highlights this complexity, as the initial presentation suggested a potential lymphoproliferative disorder such as lymphoma, yet the ultimate diagnosis was sarcoidosis. Diagnosis relies on clinical presentation, histopathology, and excluding alternative diagnoses. Initial evaluations should include comprehensive exposure history, ruling out infections (mycobacteria, fungi, parasites, histoplasmosis), and lymphoproliferative diseases. According to guidelines from the American Thoracic Society and the British Thoracic Society, screening for extrapulmonary involvement typically involves eye exams, serum tests (creatinine, alkaline phosphatase, calcium), complete blood count (CBC), and electrocardiogram (ECG), with additional tests as needed [7,8]. Lymph node biopsy is reserved for cases with mediastinal/hilar lymphadenopathy where tissue sampling is necessary [8]. Currently, endobronchial ultrasound (EBUS)-guided lymph node sampling is preferred over transbronchial lung biopsy in pulmonary sarcoidosis [8]. Elevations of serum ACE are highly suggestive of the disease. The study conducted by Groen-Hakan et al. indicates that soluble interleukin-2 receptor (sIL-2R) emerges as a valuable diagnostic marker for detecting sarcoidosis in patients with uveitis, exhibiting slightly higher diagnostic efficacy in comparison to ACE [9]. 

Sarcoidosis commonly presents with symptoms such as cough, dyspnea, wheezing, and chest pain, though auscultation is usually normal and chest X-rays show bilateral hilar adenopathy in 50% to 85% of cases [10,11]. A cross-sectional and observational study conducted in China by Cheng-Wei Li et al. found that 40.6% of patients with pulmonary sarcoidosis had extrapulmonary involvement, with extrathoracic lymph nodes and skin being the most commonly affected sites [12]. Skin involvement occurs in 25% to 35% of patients with sarcoidosis, presenting with various morphologies including papules, nodules, plaques, hypopigmented patches, subcutaneous nodules, ichthyosis, ulcers, pustules, erythroderma, and localized alopecia [13,14]. Lesions are generally multiple, erythematous and brownish, or violaceous in color, and frequently do not cause symptoms, although each type of lesion has different clinical characteristics [15]. 

Peripheral lymphadenopathy is observed in 10%-20% of cases, most commonly affecting cervical or supraclavicular sites. Inguinal, axillary, epitrochlear, or submandibular lymph node involvement is also possible [16]. However, generalized diffuse lymph node involvement in both the abdomen and chest is infrequent. A lymphoproliferative disorder should always be ruled out when generalized lymphadenopathy is present. Various studies have highlighted the difficulty in differentiating lymphoproliferative disease from sarcoidosis, as both can present with nonspecific B-symptoms. The incidence of lymphoma in patients with sarcoidosis is reported to be 5.5 to 11 times higher, indicating that both diseases can occur concurrently or sequentially, emphasizing the need to exclude lymphoproliferative disorders [2,4]. F-fluorodeoxyglucose (FDG) uptake on Positron emission tomography (PET) scans in sarcoidosis patients is nonspecific and can mimic malignancies such as lymphoma and diffuse metastatic disease, making lymph node biopsy crucial [17]. The presence of noncaseating granulomas supports a diagnosis of sarcoidosis, while the absence of lymphoma confirms the exclusion of lymphoproliferative disorders. Noncaseating granulomas have been noted across a spectrum of malignancies, including lymphomas, testicular, breast, lung, and head-and-neck cancers, with reported incidences ranging from 0.7% to 13% [18]. In particular, sarcoidosis-like granulomas were identified in 14% of individuals with Hodgkin’s lymphoma and 7% with non-Hodgkin’s lymphoma [19,20]. Therefore, the presence of granulomas on biopsy does not invariably confirm a diagnosis of sarcoidosis. It is imperative to consider other potential differential diagnoses, as highlighted by the aforementioned studies. The five-year survival rates were 57.4% for patients solely with lymphoma (diffuse large B cell lymphoma (DLBCL)) compared to 70% for patients with both lymphoma and sarcoidosis [3]. Notably, large B-cell lymphoma does not show a better prognosis when associated with sarcoidosis. Therefore, clinicians should always be aware of the potential risk of lymphoma in sarcoidosis patients presenting with new or enlarging lesions, asymmetrical nodes, or constitutional symptoms. In our case, the absence of lymphoid malignancy on flow cytometry, as well as the negative results for a malignant lymphoproliferative disorder, supported the diagnosis of sarcoidosis.

## Conclusions

This case highlights the diagnostic challenges of sarcoidosis, particularly when it presents with generalized lymphadenopathy and hypercalcemia, but without obvious pulmonary symptoms. Given its broad spectrum of clinical manifestations and the absence of specific diagnostic tests, differentiating sarcoidosis from other serious conditions, such as lymphoproliferative diseases, is crucial. In this case, the patient’s extensive lymphadenopathy and multiple pulmonary nodules raised concern for metastatic disease. However, a thorough diagnostic workup, including imaging, cervical lymph node biopsy with flow cytometry, and the exclusion of malignancies, ultimately confirmed sarcoidosis. The importance of a comprehensive, multidisciplinary approach in evaluating hypercalcemia and generalized lymphadenopathy is underscored, especially in complex cases where sarcoidosis must be considered in the differential diagnosis. This is particularly relevant due to its associations with hypercalcemia, renal dysfunction, and extrapulmonary involvement. While the presence of noncaseating granulomas on biopsy is a key indicator of sarcoidosis, clinicians must remain vigilant in ruling out other potential diagnoses, especially malignancies, as granulomas can also be seen in certain lymphomas and other granulomatous diseases.

In this case, the patient’s calcium levels and kidney function improved after conservative management of hypercalcemia. Without identifying and treating the underlying cause, the risk of recurrence and readmission with similar issues would have been high. Early diagnosis and appropriate treatment, particularly with corticosteroids (prednisone), were crucial in improving outcomes. Prednisone, a key treatment for sarcoidosis with hypercalcemia and extrapulmonary involvement, led to the resolution of hypercalcemia and improvement in acute kidney injury. This case highlights the importance of a comprehensive diagnostic approach to avoid misdiagnosis and ensure effective management of sarcoidosis. Timely diagnosis and targeted treatment can significantly improve patient outcomes and prevent complications from untreated disease.
